# Characterization of donor and recipient CD8+ tissue-resident memory T cells in transplant nephrectomies

**DOI:** 10.1038/s41598-019-42401-9

**Published:** 2019-04-12

**Authors:** Kitty de Leur, Marjolein Dieterich, Dennis A. Hesselink, Odilia B. J. Corneth, Frank J. M. F. Dor, Gretchen N. de Graav, Annemiek M. A. Peeters, Arend Mulder, Hendrikus J. A. N. Kimenai, Frans H. J. Claas, Marian C. Clahsen-van Groningen, Luc J. W. van der Laan, Rudi W. Hendriks, Carla C. Baan

**Affiliations:** 1 Department of Internal Medicine, Division of Nephrology and Transplantation, Erasmus MC, University Medical Center Rotterdam, Rotterdam, The Netherlands; 2 Department of Surgery, Division of HPB & Transplant Surgery, Erasmus MC, University Medical Center Rotterdam, Rotterdam, The Netherlands; 3 Department of Pulmonary Medicine, Erasmus MC, University Medical Center Rotterdam, Rotterdam, The Netherlands; 4 Department of Pathology, Erasmus MC, University Medical Center Rotterdam, Rotterdam, The Netherlands; 5 Department of Immunohematology and Blood Transfusion, Leiden University Medical Center, Leiden, The Netherlands

## Abstract

Tissue-resident memory T (T_RM_) cells are characterized by their surface expression of CD69 and can be subdivided in CD103+ and CD103− T_RM_ cells. The origin and functional characteristics of T_RM_ cells in the renal allograft are largely unknown. To determine these features we studied T_RM_ cells in transplant nephrectomies. T_RM_ cells with a CD103+ and CD103− phenotype were present in all samples (*n* = 13) and were mainly CD8+ T cells. Of note, donor-derived T_RM_ cells were only detectable in renal allografts that failed in the first month after transplantation. Grafts, which failed later, mainly contained recipient derived T_RM_ cells. The gene expression profiles of the recipient derived CD8+ T_RM_ cells were studied in more detail and showed a previously described signature of tissue residence within both CD103+ and CD103− T_RM_ cells. All CD8+ T_RM_ cells had strong effector abilities through the production of IFNγ and TNFα, and harboured high levels of intracellular granzyme B and low levels of perforin. In conclusion, our results demonstrate that donor and recipient T_RM_ cells reside in the rejected renal allograft. Over time, the donor-derived T_RM_ cells are replaced by recipient T_RM_ cells which have features that enables these cells to aggressively respond to the allograft.

## Introduction

Over the last two decades, the presence and importance of a non-migrating subset of memory T cells surveying immune responses in non-lymphoid tissues has been recognized: the so-called tissue-resident memory T (T_RM_) cells^1,2^. Their restricted anatomical localization in combination with their effector memory phenotype enables T_RM_ cells to rapidly respond to local antigens. Today, two distinct subsets of T_RM_ cells have been identified: CD69+CD103+ and CD69+CD103− T_RM_ cells (hereafter referred to as CD103+ T_RM_ cells and CD103− T_RM_ cells, respectively)^3–6^. CD69 is a C-type lectin that was originally identified as a marker of activated T cells but is also involved in tissue retention of T_RM_ cells^2,7–9^. CD69 binds to and down-regulates the G protein-coupled receptor sphingosine 1 phosphate (S1PR1) expressed on the T cell membrane, resulting in a decreased ability of T_RM_ cells to sense the S1P gradient that promotes migration of memory T cells from the blood into peripheral tissue^7–9^. A unique T_RM_ cell gene expression profile has recently been identified by Kumar *et al*. with 31 core genes that are differentially up- or downregulated in CD69+ T_RM_ cells isolated from human lung and spleen^10^. The other recognized T_RM_ marker, CD103, is an αE integrin that binds E-cadherin which is expressed on epithelial tissues^11^. Today, no clear consensus exists about the functional differences between CD103+ and CD103− T_RM_ cells.

Although the presence and antigen specificity of T_RM_ cells is recognized in several non-lymphoid human tissues (*e.g*. skin, liver, lungs, intestine and brain), the presence and function of T_RM_ cells in the human kidney is currently unknown^4,5,12–16^. In experimental mouse models, it was demonstrated that T_RM_ cells homed to the kidney where they resided, and that this migration was promoted by TGF-β^17,18^. Otherwise, little is known about the functional properties of these kidney T_RM_ cells^19^.

Transplantation of a renal allograft is accompanied by the transfer of donor leucocytes. It is probable that these leucocytes also include donor-derived T_RM_ cells. It is, however, unknown if these cells persist after transplantation or whether they are replaced by T_RM_ cells of recipient origin. It is therefore informative to identify the donor or acceptor origin of these cells and thus the degree of chimerism. This will help us to understand if donor T_RM_ cells are present in the allograft to control local viral and bacterial responses^20^ and whether these T_RM_ cells might be enriched for graft-*versus*-host (GvH) reactive clones, as seen in intestinal transplant patients^21–23^. The presence of recipient T_RM_ cells will shed light on their potential role in alloreactivity and will show if recipient T cells differentiate into T_RM_ cells in the renal allograft.

Here, we postulate that T_RM_ cells are present in the renal allograft and that these cells are primarily from recipient origin and are capable of mounting an allo-reactive response. To this end, we used the unique tissue resource of transplant nephrectomies from immunosuppressed patients to study the presence, provenance (donor or recipient), and the effector phenotype of CD103+ T_RM_ cells (CD69+CD103+), CD103− T_RM_ cells (CD69+CD103−), and recirculating T cells not expressing the tissue retention markers CD69 and CD103 (CD69−CD103−). We performed similar control experiments with spleen cells of organ donors, a lymphoid cell population in which T_RM_ cells are known to be present^3,10^.

## Results

### T cells of donor and recipient origin are present in the renal allograft

Thirteen transplant nephrectomy specimens were studied. Patient demographics are listed in Table 1. These kidney allografts failed either acute (*n* = 4) or chronically (*n* = 9) as a result of humoral, cellular or mixed-type rejection and were removed after a mean time of 6.1 years (range: 8 days–26 years).

**Table 1:** Patient baseline characteristics.

	Recipient				Transplantation						
	Gender	Age at nephrectomy (y)	Cause of ESRD	Number of previous renal transplants	Donor type	CMV status donor*	CMV status acceptor*	Maintenance IS	Anti-rejection treatment	Time to explantation (days)	Cause of graft failure
1.	M	30	Hypertensive nephropathy	0	L	−	+	Tac, MMF, Pred	MP, IvIg, plasmaferese, Alemtuzumab	8	aTCMR2B and aABMR and pyelonephritis
2.	F	48	ADPKD	0	L	−	+	Bela, Tac**, MMF, Pred, Basiliximab	MP, Alemtuzumab	12	aTCMR2B and aABMR
3.	M	63	Hypertension nephropathy	0	D	+	+	Tac, MMF, Pred, Basiliximab	MP, IVIg	15	aTCMR 2B and aABMR
4.	F	28	GPA	1	L	+	−	Tac, MMF, Pred	IVIg, Alemtuzumab	150	aTCMR3 and aABMR
5.	M	46	ADPKD	0	L	+	−	Tac, MMF, Pred	MP	270	aTCMR3
6.	F	67	Hypertension nephropathy	0	D	−	+	None^#^	MP	390	aTCMR3 and aABMR
7.	M	71	Diabetic nephropathy	1	D	+	+	Tac, MMF	MP	2268	aTCMR3 and c-aABMR
8.	M	29	HUS	0	D	−	−	Tac, MMF	None	2340	c-aABMR
9.	M	24	FSGS	0	L	+	+	MMF, Pred, Ecu***	MP, IVIg	2520	c-aABMR
10.	M	51	Congenital hydronephrosis	0	D	−	−	Tac	None	3240	aTCMR2B and aABMR
11.	F	51	poststreptococcal glomerulo- nephritis	0	D	+	−	None^#^	None	3780	c-aABMR and c-aTCMR
12.	M	53	Hypertension nephropathy	1	L	+	+	Tac, Pred	None	4320	aTCMR3
13.	M	57	Hypertension nephropathy	0	D	+	+	Aza, Pred	None	9360	End-stage kidney

*CMV status prior to transplantation. **Switch from Belatacept to Tacrolimus due to rejection. ***No tacrolimus due to thrombotic microangiopathy. ^#^Decreased IS and restarted dialysis. aABMR = acute antibody mediated rejection; ADPKD = autosomal dominant polycystic kidney disease; aTCMR = acute T cell-mediated rejection; Aza = azathioprine; CMV = cytomegalovirus; D = deceased; Ecu = eculizumab; ESRD = end-stage renal disease; FSGS = focal segmental glomerulosclerosis; GPA = granulomatosis with polyangitis; HUS = hemolytic uremic syndrome; IS = immunosuppression; IVIg = intravenous immunoglobulin; L = living; MP = methylprednisolone; MMF = mycophenolate mofetil; Pred = prednisone; Tac = tacrolimus.

**Figure 1:**
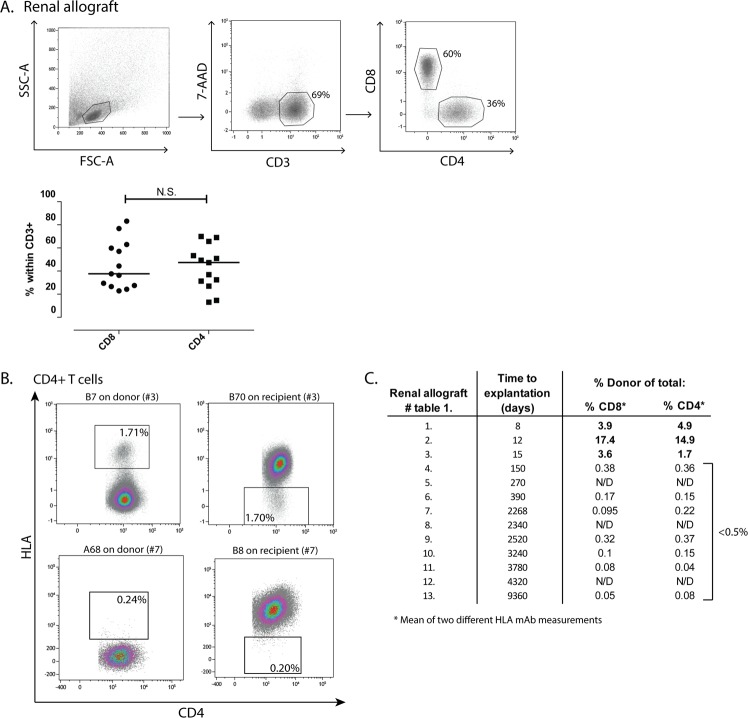
CD8+ and CD4+ T cells of donor and recipient origin are present in the renal allograft. Lymphocytes were isolated from the rejected renal allografts and subsequently stained and analysed by flow cytometry. (**A**) Gating strategy used to detect CD3+ T cells within the total viable lymphocytes, of which the CD8+ and CD4+ T cells were selected. (**B**) Typical examples of renal lymphocyte samples of patient number three and seven stained with mAb against human leukocyte antigen (HLA) class I antigens within the CD4+ T cells. Proportions of cells originating from the donor are depicted. (**C**) Table with numbers referring to the renal allografts described in Table 1, the time to explantation in days, and the proportions of donor cells detected within the CD8+ and CD4+ T cell compartment. Frequencies of the cells are presented as individual proportions with medians. HLA = human leukocyte antigen; N.S. = not significant; N/D = not determined.

After the isolation procedure, CD3+ T cells were detected in all renal allografts with a median proportion among the total viable lymphocytes of 72.1% (range: 30.1–78.5%; Fig. 1A). Of the CD3+ T cells, 37.6% (22.9–83.2%) were CD8+ and 47.4% (13.1–70.0%) were CD4+ T cells (Fig. 1A). The CD3+ T cells isolated from nephrectomy number three and four (Table 1) were capable of mounting an allogeneic response, since both the proportions of proliferating cells and the expression of the degranulation marker CD107a increased in the presence of donor antigen, while a negligible response was measured after stimulation with irradiated recipient PBMCs (Supp. Fig. 1A,B). In addition, the cells of patient number three reacted to a fully HLA mismatched 3^rd^ party and responded to donor antigen by the production of IFNγ (Supp. Fig. 1A–C).

Next, the degree of chimerism was studied in the explanted renal allografts. Conjugated HLA-mAbs were available to distinguish recipient from donor cells in ten renal lymphocyte samples. In the example depicted in Fig. 1B (upper panel), the proportions of donor cells measured by a mAb against HLA-B7 (donor positive) and a mAb against HLA-B70 (acceptor positive) resulted in similar donor proportions of 1.71% and 1.70% respectively, showing chimerism in this sample. Three out of the ten renal allografts studied were removed within the first month after transplantation (Table 1). In these renal allografts, clear populations of CD4+ and CD8+ T cells from donor origin were detected (Fig. 1C). The renal allografts removed five months or later after transplantation only contained marginal proportions (<0.5%) of donor-derived T cells, and we could not distinguish a clear positive and negative fraction in these samples as depicted in the dot plots of patient number 7 in Fig. 1B (lower panel). Thus, high proportions of donor-derived T cells were only seen in early rejection nephrectomies.

### T_RM_ cells are present in the renal allograft

**Figure 2:**
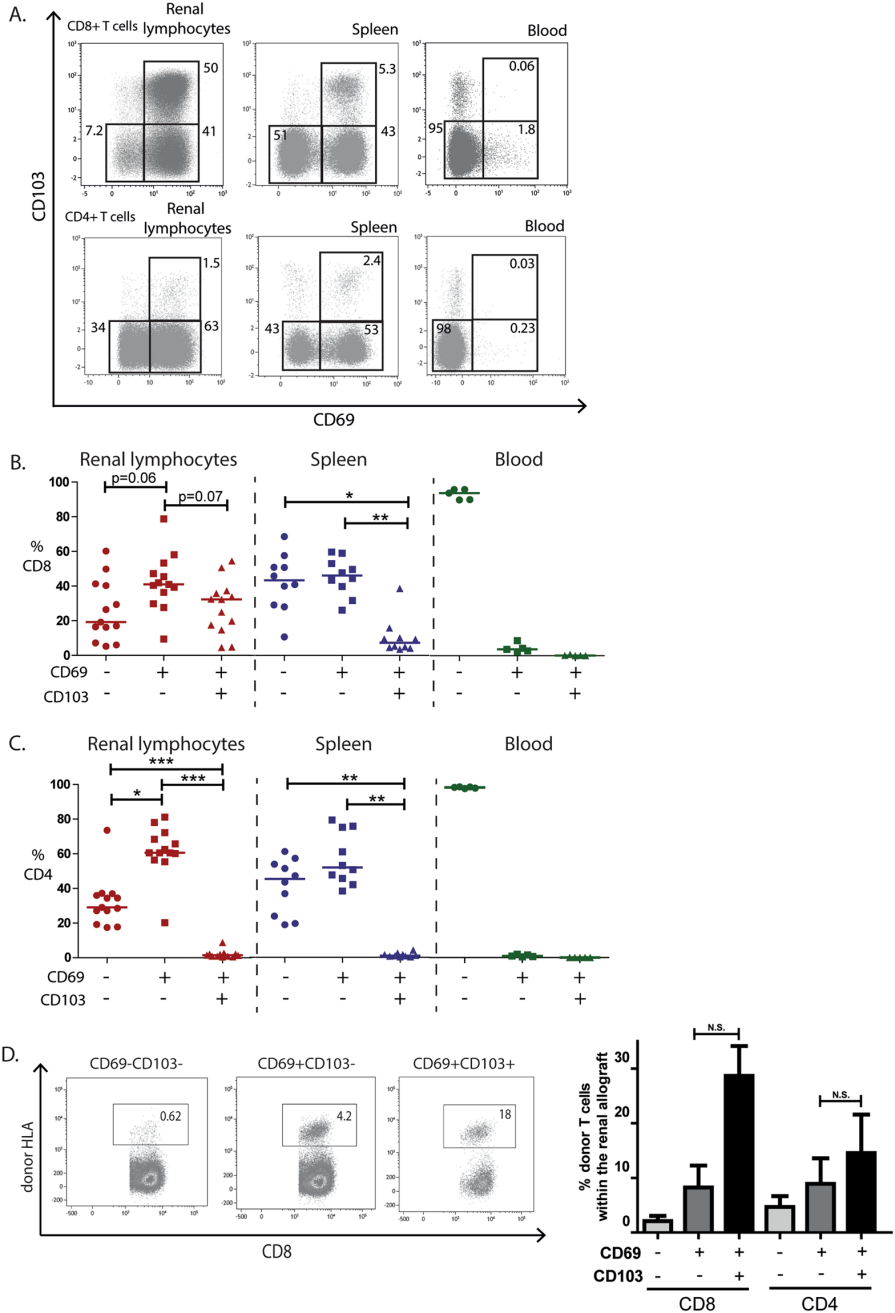
T_RM_ cells are present within CD8+ and CD4+ T cells in the renal allograft. Lymphocytes from rejected renal allografts, spleens of organ donors, and blood from healthy individuals were stained with mAb against CD69 and CD103. (**A**) Representative examples of the gating strategy of CD69 and CD103 of lymphocytes originating from the renal allograft, spleen, and blood. Proportions of the gated areas are depicted within the dot-plots. (**B**,**C**) Quantified data of the recirculating T cells (CD69−CD103−), CD103− T_RM_ cells (CD69+CD103−), and CD103+ T_RM_ cells (CD69+CD103+) subsets within the CD8+ T cell compartment (**B**) and CD4+ T cell compartment (**C**) of the renal allograft, spleen, and blood (blood *n* = 5, spleen *n* = 10, renal allograft *n* = 13). Frequencies of the cells are presented as individual proportions with medians. Significant differences were calculated and presented (*p < 0.05, **p < 0.01, ***p < 0.001). (**D**) mAb against HLA class I antigens were used to discriminate between donor and recipient lymphocytes. Typical example dot plots and quantified data of proportions of donor-derived cells within the CD103+ T_RM_ cells, CD103− T_RM_ cells and recirculating T cells are depicted of the transplant nephrectomies removed within the first month after transplantation (Table 1, patient 1 to 3). Frequencies of the positive cells are depicted within the dot plots. Frequencies of positive cells are shown as mean with the SEM (*n* = 3; N.S. = not significant).

The absence of T_RM_ cells in PBMC fractions and the presence of T_RM_ cells in the spleen was recognized in previous studies^3,10^. Subsequently we used PBMCs from healthy controls as a negative control and splenocytes of deceased organ donors as a positive control for T_RM_ cell identification. CD69+ T_RM_ cells were present in all transplant nephrectomy specimens with a median proportion of 73.2% in the CD8+ T cell compartment and 62% in the CD4+ T cell compartment (Fig. 2A). We subdivided the T_RM_ cells in CD103+ T_RM_ (CD69+CD103+) cells and CD103− T_RM_ cells (CD69+CD103−). The median proportion of CD103+ T_RM_ cells within the CD8+ T cell compartment was 32.3% (range: 4.5–54.4%) compared to 1.4% (0.7–8.8%) within the CD4+ T cell compartment. In contrast, CD103− T_RM_ cells were more evenly distributed among the CD8+ (40.9%, 9.4–78.8%) and CD4+ (60.6%, 20.2–81.2%) T cell compartments (Fig. 2B,C). As expected, minimal proportions of CD103+ T_RM_ cells and CD103− T_RM_ cells were found in peripheral blood, while both T_RM_ cell subsets were present in the spleen (Fig. 2). Similar proportions of CD4+ and CD8+ T_RM_ cells were detected in two native kidneys that were discarded for transplantation (Supp. Fig. 2). No significant differences were detected when we subdivided the CD103+ and CD103− T_RM_ cells of the transplant nephrectomies based on donor type, Banff 2017 category and time to explantation (Supp. Fig. 3A–C). Of interest might be the observation that in chronic-active antibody-mediated rejection (c-aABMR) specimens the proportion of CD103− T_RM_ cells was low (Supp. Fig. 3B). However, this observation is only based on two c-aABMR cases.

We questioned whether T_RM_ cells of donor or recipient origin were present in the kidneys explanted in the first month after transplantation (Fig. 1C). For that we stained the renal lymphocytes of the transplant nephrectomies removed within the first month after transplantation (patients 1–3, Table 1) again with antibodies recognizing either donor or recipient HLA molecules. Remarkably, donor-derived lymphocytes were most prominent within the CD103+ T_RM_ cell population, with lower levels of donor cells in the CD103− T_RM_ cells and recirculating T cell compartments (Fig. 2D). This was observed for both CD8+ and CD4+ donor T cells. These data suggest the increased ability of donor-derived CD103+ T_RM_ cells to reside in the renal allograft compared to the remaining donor-derived T cells.

Because the proportion of CD103+ T_RM_ cells was very low among the CD4+ T cells, not allowing further analysis, we focused on the CD8+ T_RM_ cells for subsequent experiments. In addition, the three renal allografts explanted within the first month after transplantation were excluded in order to study a pure population of recipient T_RM_ cells that may mediate and control the local immune response.

### Expression of T_RM_ signature genes

**Figure 3:**
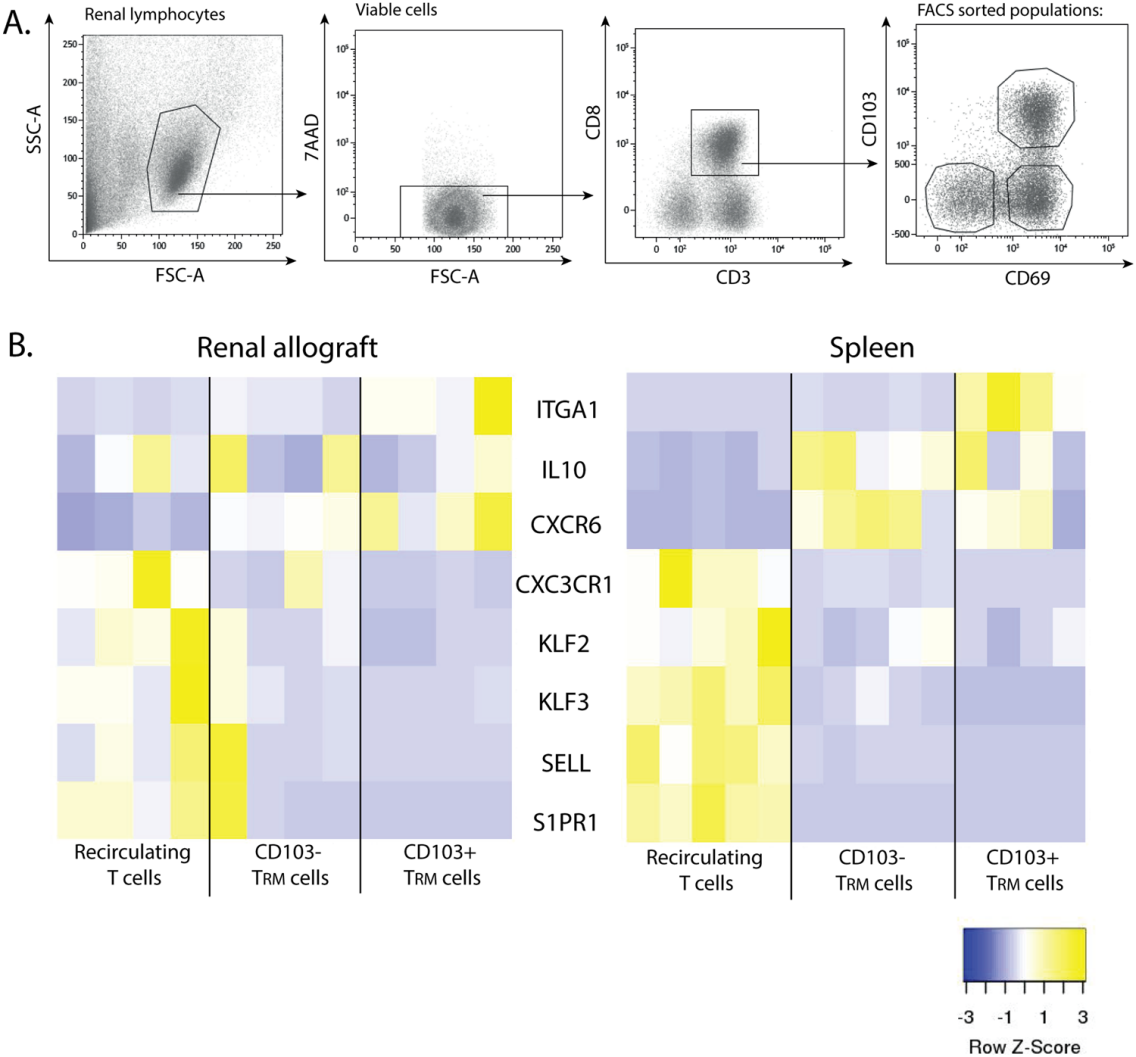
Expression of T_RM_ signature genes. (**A**) Typical example of the gating strategy used after fluorescence activated CD8+ cell sorting to obtain recirculating T cells (CD69−CD103−), CD103− T_RM_ cells (CD69+CD103−), and CD103+ T_RM_ cells (CD69+CD103+). Cells were gated by forward- and side-scatter followed by 7-AAD negative (viable) gating. (**B**) Heatmaps depicting the normalized gene expression of eight signature genes known to be upregulated (yellow) or downregulated (blue) in T_RM_ cells of the renal allograft and spleen. Spleen *n* = 5, renal lymphocytes *n* = 4.

To define the gene expression profile of the T_RM_ cells, we selected 8 genes from a set of signature genes described by Kumar *et al*. that are differentially expressed between CD69− and CD69+CD8+ T cells isolated from the spleen and lung^10^. We compared the expression of these genes in cells isolated form the renal allograft and in splenocytes in the following FACS-sorted CD8+ T cell populations: CD69+CD103+ T_RM_ cells, CD69+CD103− T_RM_ cells, and CD69−CD103− (recirculating) T cells. The gating strategy for the FACS sort experiments is depicted in Fig. 3A.

We used RT-qPCR to quantify the expression of genes that are involved in pathways that mainly control T cell migration, adhesion and activation. Separate heat maps were created of the gene expression levels of the renal allograft and spleen (Fig. 3B). Overall, two clusters were identified in the heat maps of both the renal allograft and spleen: a cluster of genes upregulated in the total T_RM_ cells (CD103+ and CD103− T_RM_ cells) and a cluster of genes downregulated in the total T_RM_ cells, compared to the recirculating T cells (Fig. 3B). The gene expression levels of the adhesion marker ITGA1 (CD49a) were especially high in the CD103+ T_RM_ cells, with lower expression levels in the CD103− T_RM_ cells and recirculating T cells. Chemokine receptor CXCR6 and cytokine IL-10 were clearly expressed at a higher level in the total T_RM_ cell subsets compared to the recirculating T cells. In both the spleen and renal allograft samples, the T cell trafficking and homing markers S1PR1, Kruppel-like transcription factor 2 (KLF2), SELL (CD62L), KLF3 and CX3CR1 were expressed at a lower level in the total T_RM_ cells compared to the expression levels in the recirculating T cells. When we clustered the FACS-sorted samples of the renal allograft and spleen, the CD103+ T_RM_ cells and CD103− T_RM_ cells clearly clustered together as opposed to the recirculating T cells (Supp. Fig. 4). In summary, the total population of T_RM_ cells can be clearly distinguished from the recirculating T cells based on their gene expression profile. In addition, similar gene expression levels were found in the different T cell subsets when comparing the renal allograft and the spleen samples.

### T_RM_ cells in the renal allograft have an effector memory phenotype

**Figure 4:**
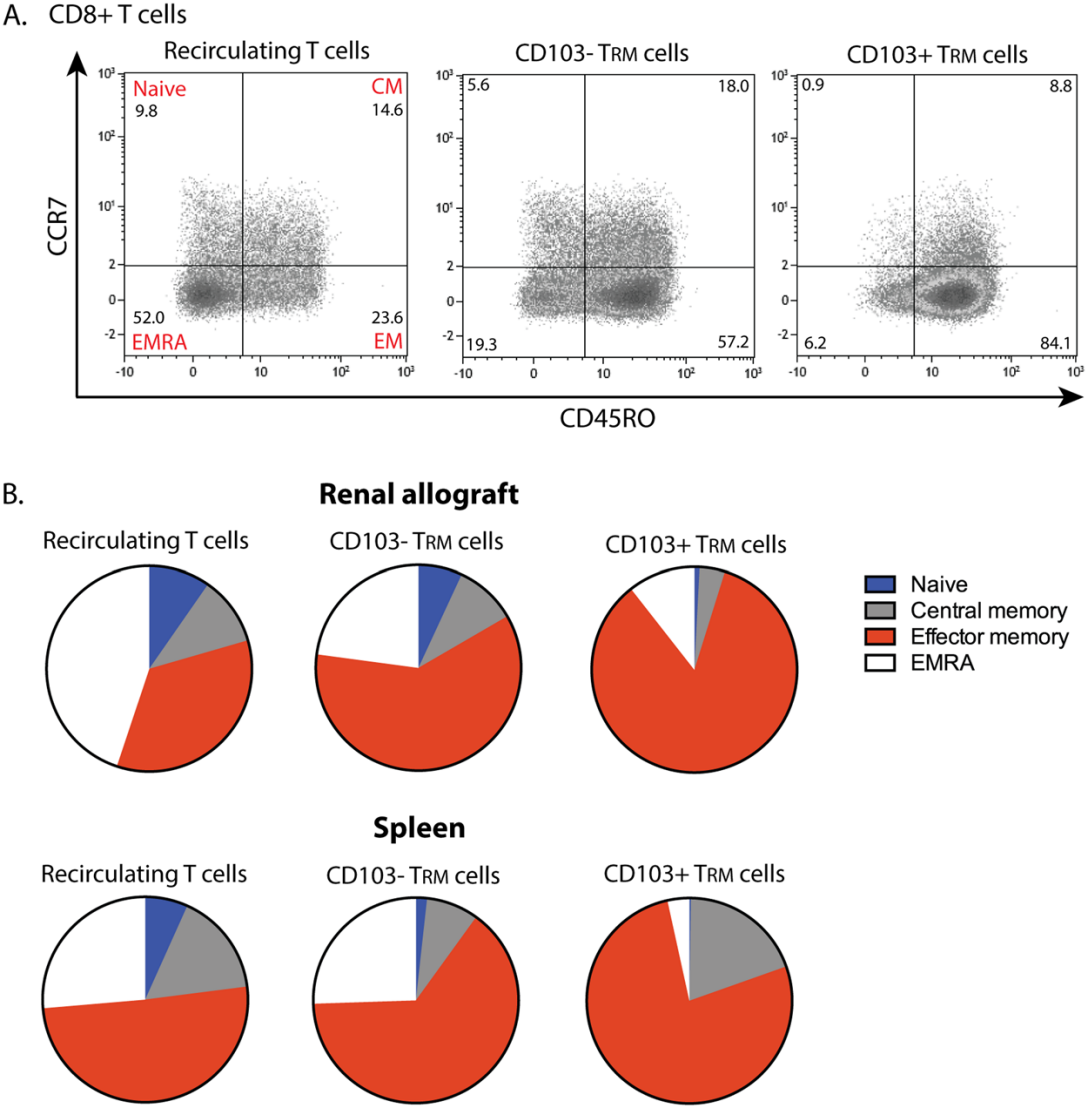
T_RM_ cells in the renal allograft have an effector memory phenotype. (**A**) Typical examples of dot plots presenting the distribution of naïve T cells (CCR7+CD45RO−), central memory T cells (CM; CCR7+CD45RO+), effector memory T cells (EM; CCR7−CD45RO+), and EMRA T cells (CCR7−CD45RO−) within the CD8+ recirculating T cells, CD103− T_RM_ cells and CD103+ T_RM_ cells of the renal allograft. Numbers within the dot plots indicate proportions of the different cell subsets. (**B**) Pie charts representing the median proportion of naïve, central memory, effector memory, and EMRA T cells within the CD8+ recirculating T cells, CD103− T_RM_ cells and CD103+ T_RM_ cells of the renal allograft and spleen (renal lymphocytes *n* = 6, spleen *n* = 8).

To demonstrate that the CD8+ T_RM_ cells found in the renal allograft have an effector phenotype we stained the cells for the surface molecules CCR7 and CD45RO. With these markers naïve (CCR7+CD45RO−), central memory (CM, CCR7+CD45RO+), effector memory (EM, CCR7−CD45RO+) and highly-differentiated effector memory (EMRA, CCR7−CD45RO−) T cells were discriminated (see Fig. 4A for a typical example). While the recirculating T cells in the renal allograft and spleen were more divergent in terms of CCR7 and CD45RO expression, the majority of the CD103− T_RM_ cells and CD103+ T_RM_ cells were CCR7−CD45RO+ and thus EM T cells (Fig. 4B). This finding is in line with previous studies in non-lymphoid tissues where the majority of T_RM_ cells was also of an EM phenotype^3,10^.

### T_RM_ cells are capable of producing effector molecules

The capacity of the cells to produce the pro-inflammatory cytokines IFNγ and TNFα was measured after polyclonal stimulation. No change in the expression and proportion of CD69+ cells before and after three hours of polyclonal T cell stimulation was observed, in contrast to an altered expression of CD69 and CD103 on the CD8+ T cells after seven days allostimulation (Supp. Figs 5 and 6). Subsequently, we compared the T_RM_ cell subsets and the recirculating T cells after stimulation for their cytokine production capacity.

**Figure 5:**
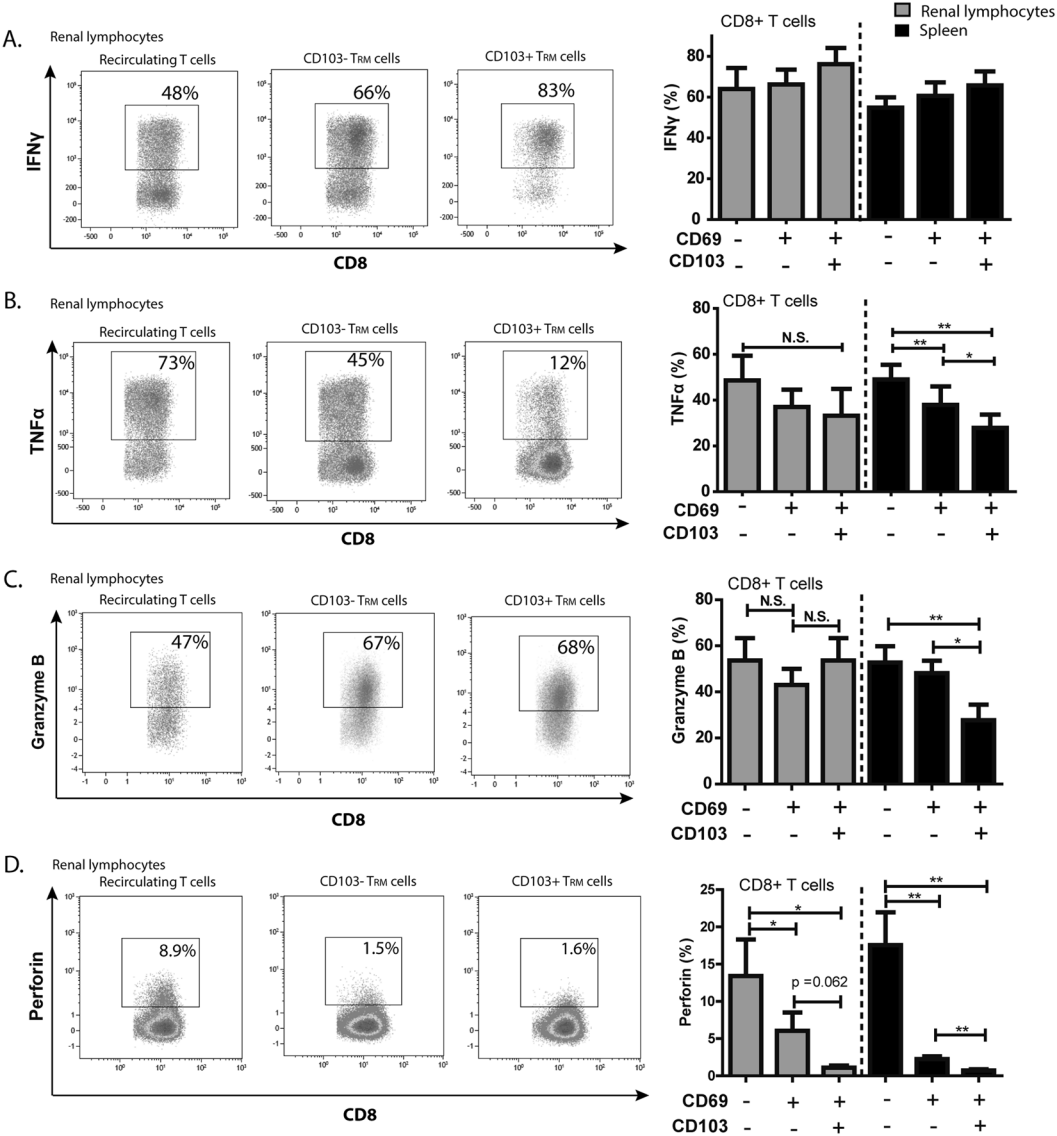
T_RM_ cells are capable of producing effector molecules. (**A**,**B**) Proportions of IFNγ (**A**) and TNFα (**B**) producing cells are depicted upon 4 hours PMA/ionomycin stimulation in the presence of monensin and brefeldin A. Cytokine proportions were measured in renal lymphocytes and splenocytes within the recirculating T cells, CD103− T_RM_ cells, and CD103+ T_RM_ cells of the CD8+ T cell compartment. (**C**,**D**) Frequencies of granzyme B (**C**) and perforin (**D**) levels were measured in the recirculating T cells, CD103− T_RM_, and CD103+ T_RM_ cells of the CD8+ T cell compartment. Frequencies of positive cells were shown as mean with the SEM (renal lymphocytes *n* = 6, spleen *n* = 10). Significant differences were calculated and depicted (N.S. = not significant, *p < 0.05, **p < 0.01).

After stimulation, all resident and recirculating CD8+ T cell subsets expressed high levels of IFNγ (Fig. 5A). A slightly higher expression in the IFNγ production capacity was observed in the CD103+ T_RM_ cells in both renal allograft and spleen compared to the recirculating and CD103− T_RM_ cell subsets (Fig. 5A). For TNFα, a different profile was found with the highest expression levels in the recirculating T cells, lower levels in the CD103− T_RM_ cells and the lowest levels in the CD103+ T_RM_ cells (Fig. 5B). Within the spleen, the differences in TNFα proportions were significantly different between the different subsets (Fig. 5B). Highly effector CD8+ T cells that were concurrently positive for IFNγ and TNFα were present in both the renal CD103− and CD103+ T_RM_ cells (Supp. Fig. 7).

To determine the degranulation capacity of the different CD8+ T_RM_ cell subsets, intracellular granzyme B and perforin expression were measured (Fig. 5C,D). In the renal allograft, the intracellular granzyme B levels did not significantly differ between the recirculating T cells, the CD103− T_RM_ cells and CD103+ T_RM_ cells (Fig. 5C). The granzyme B levels within the spleen were significantly lower in the CD103+ T_RM_ cells compared to both the recirculating T cells and the CD103− T_RM_ cells. With regard to the intracellular levels of perforin, the same trend between the different subsets was found in the renal allograft samples compared to the spleen samples (Fig. 5D). The perforin levels were significantly lower in the CD103+ T_RM_ cells compared to the recirculating T cells (Fig. 5D). Together, these data show that T_RM_ cells, but also the recirculating T cells, are capable of mounting and effector response.

## Discussion

**Figure 6:**
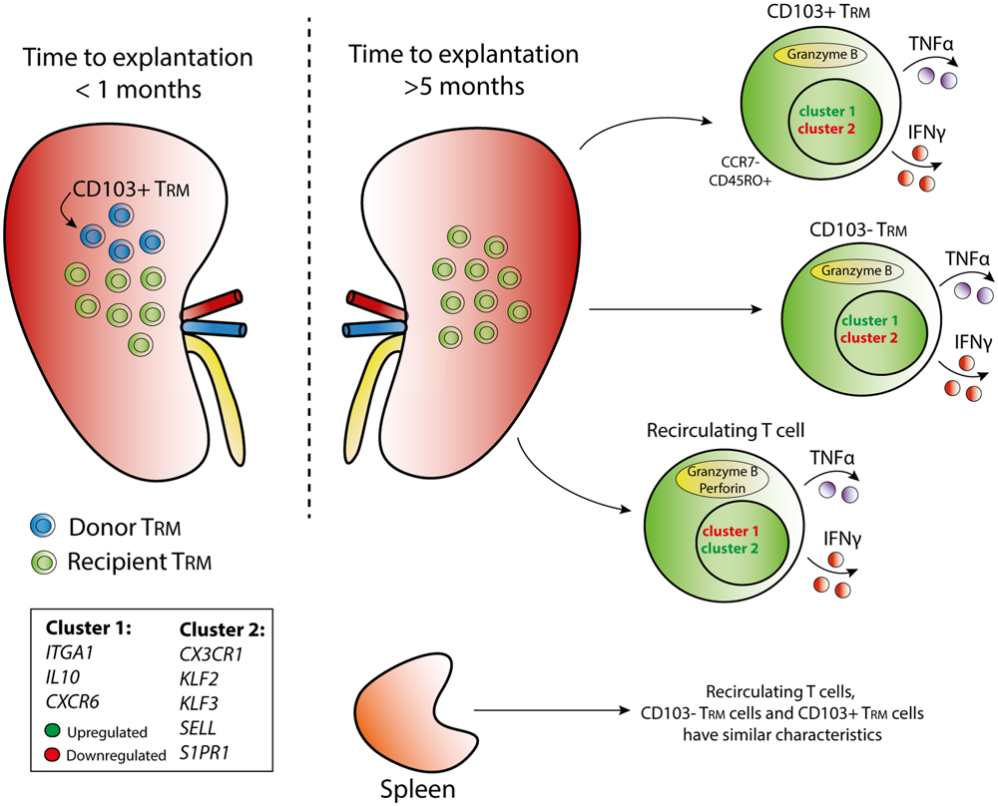
Schematic overview of T_RM_ cell characteristics in the renal allograft. Distribution of donor-derived and recipient-derived tissue-resident memory (T_RM_) cells and the phenotypic and functional characteristics of the recipient-derived T_RM_ cells in the explanted renal allograft are depicted in a schematic overview. Cluster one and cluster two indicate T_RM_ core genes of which the expression was measured. Cluster one consists of genes involved in T cell activation (*ITGA1*, *IL10*, and *CXCR6*) and cluster two consists of genes involved in T cell migration (*CX3CR1*, *KLF2*, *KLF3*, *SELL*, *S1PR1*).

In this study, we demonstrate that (1) T cells with a resident memory phenotype and gene expression profile are present in the renal allograft, (2) T_RM_ cells in the renal allograft have strong immunostimulatory capacity, and (3) the donor-derived cells present are mainly CD103+ T_RM_ cells and are replaced by recipient-derived T cells within five months after transplantation. An overview of these findings is depicted in Fig. 6.

No major differences were found in gene expression signature and functional profiles between the CD103+ T_RM_ and CD103− T_RM_ cells, highlighting that these two T_RM_ cell subtypes have comparable characteristics. Previous studies report that CD103+ T_RM_ cells reside more predominantly within the barrier tissues while CD103− T_RM_ cells are more common within non-barrier tissues^18^. In transplantation, CD8+CD69−CD103+ T cells are involved in the effector mechanism of chronic and acute renal allograft rejection^24–27^. In addition, in urinary samples, the mRNA levels of CD103 predicted acute renal allograft rejection^28^. However, in the context of kidney transplantation the exact difference in CD103+ and CD103− T_RM_ cells is unknown and of interest for further analysis.

The majority of the T_RM_ cells detected in the renal allograft were of an effector memory phenotype. Therefore, these cells have been antigen challenged and are able to rapidly exert immunological responses. Both the CD103+ and CD103− T_RM_ cell subsets had the capacity to produce TNFα, a cytokine involved in, among others, the activation of endothelial cells, thereby attracting other T cells to the site of inflammation^29^. The capacity of the T_RM_ cells to produce IFNγ and the presence of preloaded granzyme B positive granules underlines their cytotoxic phenotype. Low levels of perforin were measured in the T_RM_ cells which is in line with previous studies^30,31^. In these studies, perforin was rapidly upregulated upon antigen stimulation, delineating a dynamic process^30,32^. Here we found that T_RM_ cells present in renal allografts are potentially harmful cells that might contribute to the process of allograft rejection. However, additional experiments need to clarify the exact roles of the T_RM_ cell subsets in the alloimmune response and show causality. For future studies, it would be of interest to include the number of cells per gram tissue. The functional profiles of the T_RM_ subtypes found in the renal allograft were comparable to those found in the T_RM_ subsets residing in the spleen. This supports that we have identified T_RM_ cells in the renal allograft.

Memory T cell signalling is still occurring under immunosuppression because these cells are less reliant on co-stimulatory signals^33–36^. For this reason, we can hypothesize about the long lasting persistence of T_RM_ cells within the renal allograft. Also, the distribution of immunosuppressive agents into the tissue may influence the presence of T_RM_ cells. For instance, alemtuzumab depletes circulating CM T cells in leukemic cutaneous T cell lymphoma patients without completely compromising the immune response to infection, since the skin resident memory cells are spared^37^. The samples analysed in this study are a heterogeneous group with different types of end-stage immunological transplant failure. Further study on the differentiation and function of T_RM_ cells upon transplantation, would benefit from similar studies on cells harvested from healthy renal tissue or grafts undergoing an evolving rejection, which for obvious reasons is not possible in transplant recipients. The best alternative for healthy renal tissue would be the use of kidneys discarded for transplantation. Our first findings showed the presence of T_RM_ cells in these kidneys with comparable frequencies of CD103− and CD103+ T_RM_ cells as found in the transplant nephrectomies.

The results of our study reveal that donor-derived lymphocytes are replaced by their recipient counterparts within five months after transplantation and that the recipient-derived T cells differentiate locally towards a resident memory phenotype. The rapid presence of recipient T_RM_ cells in these specimens sheds light on the important role of this cell type in the process of alloreactivity. The repopulation by recipient lymphocytes has also been observed in lung and intestinal allografts^38,39^. The prolonged retention of the CD103+ T_RM_ cells compared to the other donor-derived T cells supports that this subtype of T_RM_ cells is highly capable to bind to epithelial cells of the renal allograft (Fig. 6). The retention of donor-derived T_RM_ cells might have a protective role in the renal allograft since irradiation of donor cells in rodent models resulted in rejection of liver transplants^40,41^. Also, in visceral transplant patients, T cell chimerism is observed in the absence of graft-*versus*-host disease (GVHD)^21^. Donor-derived cells with graft specific TCR clones are even thought to slow down the constant threat of recipient-derived T cells, illustrating that the balance between graft-*versus*-host (GvH) and host-*versus*-graft (HvG) clones *in situ* influences lymphocyte turnover and development of rejection^22^. A rapid clearance of the donor-derived cells may thus contribute to the rejection of the renal allograft. The low IFNγ-producing response of the renal lymphocytes to recipient PBMCs that we measured might be due to the mixed population of donor and recipient cells within the renal lymphocytes. This observation might be a reflection of the potential GvH response which may also explain a lower response to the donor cells, in line with the findings of Zuber *et al*.^22^. For future experiments, it is of interest to compare the GvH and HvG T cell balance in protocol biopsies at different time points after transplantation and compare this balance between patients with and without rejection. In addition to the response to the allograft, T_RM_ cells also recognize and clear pathogens. Therefore, it is tempting to speculate that the T_RM_ cells are both friends and foes to the renal allograft. Virus-specific T_RM_ cells are known to be able to quickly exert their effector function within the peripheral tissue, and thus the T_RM_ cells present in the renal allograft may also exert this function^20,42^. Therefore, the precise contribution of donor and recipient T_RM_ cells to alloreactivity is of high interest for future studies.

In conclusion, our results demonstrate that T_RM_ cells are present in the human renal allograft and that donor-derived T_RM_ cells are replaced within the first months after transplantation by recipient T_RM_ cells, which have the capacity to aggressively respond to the allograft. Understanding the potentially destructive or protective roles of the T_RM_ cells in the renal allograft is of high interest to enhance renal transplant outcomes.

## Materials and Methods

### Study population

Thirteen transplant nephrectomy specimens and two kidneys that were discarded for transplantation were studied. The characteristics of the patients from whom the transplant nephrectomies were derived are described in Table 1. Of these nephrectomies, the pathological features corresponding to the type of renal allograft rejection were classified according to the Banff’17 classification^43^. In this study, residual material previously used for histopathological diagnosis was analysed. Residual materials were used in accordance with non-WMO compliant research that is regulated by the Dutch Code of Conduct (Federa). Splenocytes were obtained from deceased organ donors and peripheral blood mononuclear cells (PBMCs) were from healthy controls. The Medical Ethical Committee of the Erasmus MC, University Medical Center, approved this work (MEC-2010-022). All experiments were performed in accordance with relevant guidelines and regulations as described by our institution. All patients gave written informed consent. No organs were procured from (executed) prisoners.

### Isolation of lymphocytes

Half of the renal allograft (cortex and medulla) was processed towards a single cell suspension, the other half was used for routine diagnostic assessment. The renal allograft was thoroughly rinsed with PBS to remove peripheral cells and afterwards dissected into small pieces (<0.5 cm^3^) and incubated for 60 minutes at 37 °C with 1.1 mg/ml collagenase IV (Serva, Heidelberg, Germany). The collagenase treatment was stopped by the addition of heat-inactivated fetal bovine serum with an end concentration of 10%. The tissue suspension was filtered through different sieves up to a 100 μm sieve (Greiner Bio-One, Kremsmünster, Austria) followed by a Ficoll-Paque Plus procedure (GE healthcare, Uppsala, Sweden). Isolated cells were stored at −190 °C until further use. Afterwards, cells were thawed and analysed by flow cytometry and reverse transcription-qPCR (RT-qPCR) to determine their phenotype and gene expression profile.

Human splenocytes were disrupted and filtered through a 70 μm cell strainer (Greiner Bio-one, Alphen a/d Rijn, The Netherlands) to obtain a single-cell suspension. Ficoll-paque (Amersham Pharmacia Biotech, Uppsala, Sweden) density gradient was used to collect mononuclear cells. Human PBMCs were isolated with the Ficoll-paque density gradient method.

### Flow cytometric and functional analysis

Renal lymphocytes, splenocytes and PBMCs were stained with the following antibodies to characterize the T_RM_ phenotype: CD3 brilliant violet 510 (BV510; Biolegend, San Diego, CA, USA), CD8 Allophycocyanin-Cy7 (APC-Cy7; Biolegend), CD4 fluorescein isothiocyanate (FITC; BD, Franklin Lakes, New Jersey, USA) CD69 brilliant violet 421 (BV421; BD), CD103 phycoerythrin-cyanine7 (PE-Cy7; Biolegend). Viability was measured with the live-dead marker 7-aminoactinomycin (7-AAD; BD). FACS flow enriched with bovine serum albumin was used to wash the cells and block a-specific antibody interactions.

The donor or acceptor origin of the cells was determined with monoclonal antibodies (mAb) directed against human leukocyte antigen (HLA) class I antigens of the donor or acceptor. mAb used are listed in Supplemental Table 1, and were developed at Leiden University Medical Centre. We performed single labelling with either an antigen for which solely the donor was positive or solely the acceptor was positive.

The cytotoxic potential of T_RM_ cells was analysed by measuring intracellular granzyme B and perforin. Cells were stained as described above for T_RM_ cells. Afterwards, the cells were immediately fixed with FACS lysing solution (BD) and permeabilized with PERM II (BD). Subsequently, cells were stained intracellularly with granzyme B Allophycocyanin (APC) (Biolegend) and perforin phycoerythrin (PE; Biolegend).

The cytokine-producing capacity of the cells was measured after the cells were stimulated for four hours with 50 ng/ml phorbol myristate acetate (PMA) and 1 μg/ml ionomycin (Sigma-Aldrich, St. Louis, MO, USA) at 37 °C. Monensin and Brefeldin A (GolgiStop and GolgiPlug, BD Biosciences, Franklin Lakes, NJ, USA) were used to promote intracellular accumulation of the cytokines. PMA/ionomycin stimulation was stopped by adding Ethylene-diamine-tetra-acetic acid. Subsequently, cells were stained with the T_RM_ surface staining mixture and fixed and permeabilized as described above. The following mAb were used to measure cytokine production: interferon-γ (IFNγ) FITC (BD Biosciences), and tumour necrosis factor-α (TNFα) PE (Biolegend). Cell samples were measured on the FACSCanto II (BD) and analysed with Kaluza Analysis 1.5a software (Beckman Coulter, Brea, CA, USA).

### Allogeneic reactivity

Mixed lymphocyte reactions (MLR) were performed in order to study T cell-reactivity of the renal lymphocytes following stimulation with donor cells, 3^rd^ party cells, or recipient PBMCs. Renal lymphocytes were thawed and labelled with carboxyfluorescein succinimidyl ester (CFSE; Molecular Probes^®^, Leiden, The Netherlands). Next, the renal lymphocytes were stimulated at 5 × 10^4^ cells per well with irradiated donor cells, 3^rd^ party cells, or recipient PBMCs (40 Gray) from which the CD3+ T cells had been depleted with magnetic-activated cell sorting (MACS). The cells were cultured at a ratio 1:1 in human culture medium (HCM) and after 7 days the CFSE dilution was measured on the FACSCanto II, indicating the amount of proliferation. Degranulation was measured by APC-labelled anti-CD107a (BD).

### IFNγ ELISPOT

The frequencies of IFNγ producing cells upon stimulation with donor cells, 3^rd^ party cells, or recipient PBMCs were measured with an Enzyme-Linked ImmunoSpot (ELISPOT) assay (U-CyTech Biosciences, Utrecht, The Netherlands). Briefly, anti IFNγ coated plates (U-CyTech Biosciences), were seeded with 100,000 renal lymphocytes and 100,000 irradiated donor cells, 3^rd^ party cells or recipient PBMCs. Upon overnight incubation, plates were washed and incubated with biotinylated anti-human IFNγ detection antibody and streptavidin-HRP conjugate followed by the addition of AEC substrate (U-CyTech Biosciences). Spots were analysed using the ELISPOT reader (Bioreader^®^-600V, BIO-SYS GmbH, Karben, Germany).

### Cell sorting of T_RM_ cells and RT-qPCR analysis

To determine the expression levels of a set of T_RM_ key genes of the CD103+ and CD103− T_RM_ cells and CD69−CD103− recirculating T cells, we performed RT-qPCR analysis on FACS-sorted, pure (>95%) T cell populations. Renal lymphocytes and splenocytes were sorted with the BD-FACSAria II SORP^TM^. Subsequently, cells were pelleted and snap frozen and stored at −190 °C until further use.

RNA was isolated with the RNeasy Micro Kit (Qiagen, Hilden, Germany) for the collection of high-quality RNA. Total RNA was subsequently reverse transcribed with oligo-dT. We used RT-qPCR to quantify the amount of *ITGA1*, *IL10*, *CXCR6*, *CX3CR1*, *KLF2*, *KLF3*, *SELL*, *S1PR1*, and the housekeeping gene *glyceraldehyde 3-phosphate dehydrogenase* (*GAPDH*). Assay-on-demand products for the detection and quantification of the different genes were used and are listed in Supplemental Table 2 (ThermoFisher, Waltham, MA, USA). The amount of each target gene was quantified by measuring the threshold cycle (Ct), which was transformed on a TaqMan Real-Time PCR system to the number of cDNA copies (2^(40-Ct)^). The relative concentrations of the analysed genes were normalized to the relative concentration of the housekeeping gene GAPDH present in each sample. Heatmapper software was used to cluster the cell-sorted samples based on the expression of the above described genes^44^.

### Statistical analysis

Statistical analyses were performed using GraphPad Prism 5 software (GraphPad Software; San Diego, CA, USA). Differences between paired groups were analysed with the Wilcoxon signed-rank test. A two-tailed *p*-value of < 0.05 was considered statistically significant.

## Supplementary information

Supplemental figures dataset 1

## Data Availability

All data generated or analysed during this study are included in this published article and the Supplementary Information File.
