# Supplementary material for: Characterization of donor and recipient CD8+ tissue-resident memory T cells in transplant nephrectomies
Source: Sci Rep. 2019 Apr 12;9:5984. doi: 10.1038/s41598-019-42401-9 (PMC6461670; doi:10.1038/s41598-019-42401-9)
Supplement: Supplementary file 1 — Supplemental figures dataset 1 [file 41598_2019_42401_MOESM1_ESM.pdf]

Supplemental Figures and Tables

Characterization of donor and recipient CD8+ tissue-resident memory T cells in transplant nephrectomies

Kitty de Leur[1,2]*, Marjolein Dieterich[1], Dennis A. Hesselink[1], Odilia B.J. Corneth[3], Frank J.M.F. Dor[2], Gretchen N. de Graav[1], Annemiek M.A. Peeters[1], Arend Mulder[5], Hendrikus J.A.N. Kimenai[2], Frans H.J. Claas[5], Marian C. Clahsen-van Groningen[4], Luc J.W. van der Laan[2], Rudi W. Hendriks[3], Carla C. Baan[1]

[1]Department of Internal Medicine, Division of Nephrology and Transplantation [2]Department of Surgery, Division of HPB & Transplant Surgery, [3]Department of Pulmonary Medicine, [4]Department of Pathology, Erasmus MC, University Medical Center Rotterdam, The Rotterdam Transplant Group, The Netherlands, [5]Department of Immunohematology and Blood Transfusion, Leiden University Medical Centre, The Netherlands

Corresponding author:
*Kitty de Leur, MSc
ORCID identifier: 0000-0002-4550-9033
Email: k.deleur.1@erasmusmc.nl
Address: Dept. of Internal Medicine, Division of Nephrology and Transplantation
Erasmus MC, University Medical Centre Rotterdam, Room Na-522,
Dr. Molewaterplein 40, 3015 GD Rotterdam

Name	HLA specificity	Type
SN607d8	A2, A68	IgG1, κ
VTM1F11	B27	IgG1, κ
SN230G6	A2	IgG1, λ
OK2F3	A3	IgM, κ
GV5D1	A1, A23, A24	IgG1, λ
BVK1F9	B8	IgG1, κ
MUL6D1	A11	IgM, κ
DK7C11	B44	IgG1, κ
HDG8D9	B35	IgG1, λ
OK8F12	B70	IgG1, κ

Supplemental table 1. List of human monoclonal anti-HLA antibodies used in this study. All monoclonal antibodies were labeled to the fluorochrome alexa-488

Gene	ID
GAPDH	Hs99999905.m1
ITGA1/CD49a	Hs00235006_m1
ITGAE/CD103	Hs01025372_m1
CXCR6	Hs00174843_m1
SELL/CD62L	Hs00174151_m1
KLF3	Hs00610885_m1
KLF2	Hs00360439_g1
S1PR1	Hs00173499_m1
IL10	Hs00174086.m1
CX3CR1	Hs00365842_m1

Supplemental table 2. Primers used for gene expression assays

Supplemental figure 1.

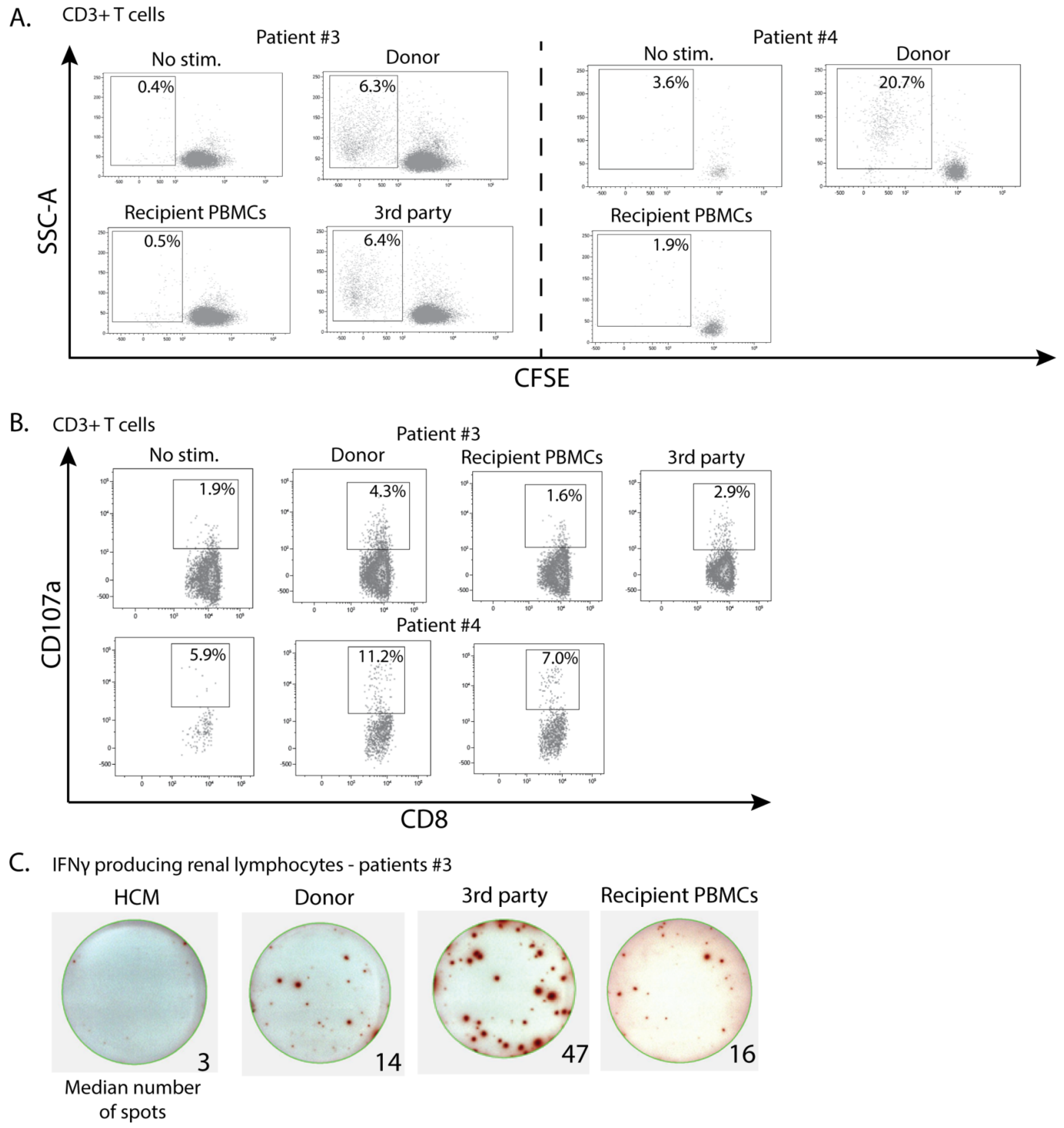

D. HLA-typing patient #3

Recipient:	A3 A23(9)	B8 B72(70)	DR17(3) DR7
Donor:	A3 A1	B8 B7	DR17 (3) DR4
3rd party:	A2 A33(19)	B44(12) B58(17)	DR11(5) DR13(6)

HLA-typing patient #4

Recipient:	A3 A25(10)	B7 B44(12)	DR15(2) DR4
Donor:	A1	B8	DR3 DR4

Supplemental figure 1.

Renal lymphocytes do have specificity for donor cells

(A) Renal lymphocytes of transplant nephrectomy number three and four (Table 1, Fig. 1C) were labelled with CFSE and stimulated with only human culture medium (HCM), donor cells, 3rd party cells, or recipient PBMCs for seven days. Afterwards, the proportion of proliferating cells was measured by gating the CFSE negative T cell fraction. (B) Degranulation of the renal lymphocytes after seven days of stimulation with HCM, donor cells, 3rd party cells, or recipient PBMCs was measured by measuring the CD107a+ T cell fraction. (C) Numbers of IFNγ producing cells were measured with the ELISPOT assay after stimulating the renal lymphocytes with HCM, donor cells, 3rd party cells, or recipient PBMCs. (D) HLA-typing of the renal allograft recipients, donor and 3rd party cells used for the mixed lymphocyte reaction and ELISPOT assay. The number of renal lymphocytes of patient four was not sufficient to include a 3rd party control and to perform an ELISPOT assay.

Supplemental figure 2.

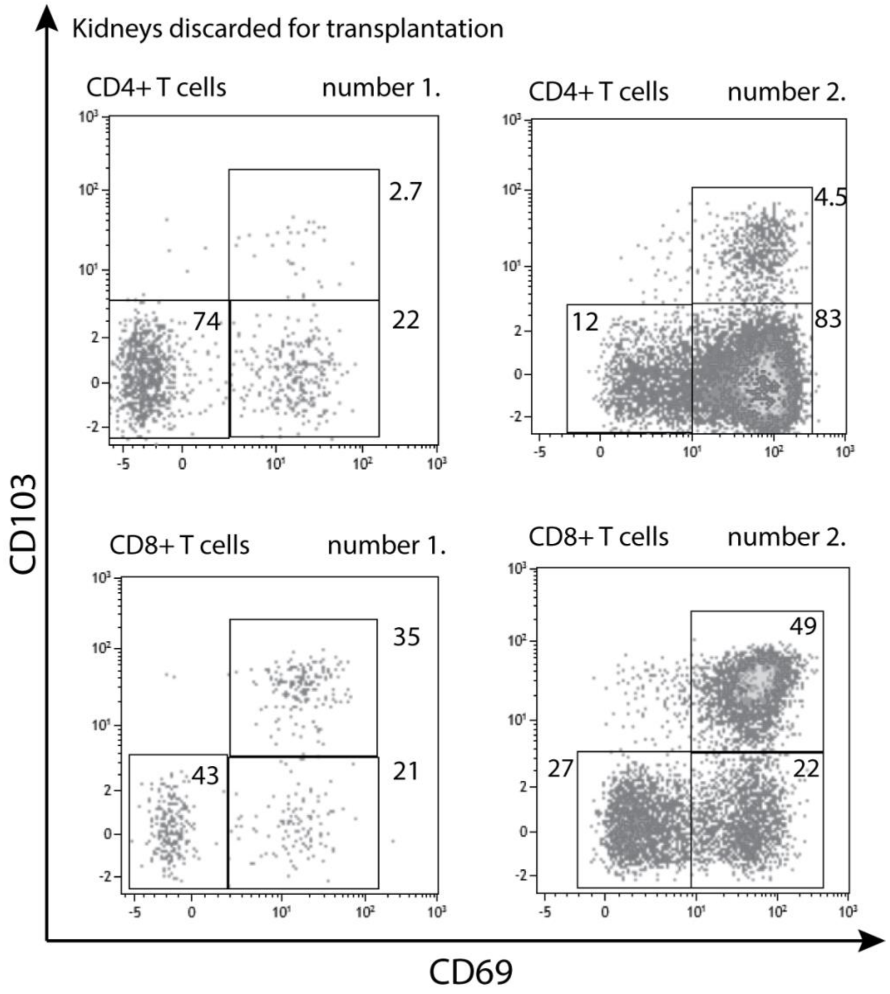

Supplemental figure 2.

T_{RM} cells are present in two kidneys that were discarded for transplantation

Lymphocytes from two kidneys that were discarded for transplantation were stained with mAb against CD69 and CD103. Frequencies of CD69+ and CD103+ T cells within the CD4+ and CD8+ T cell compartment are depicted within the figure.

Supplemental figure 3.

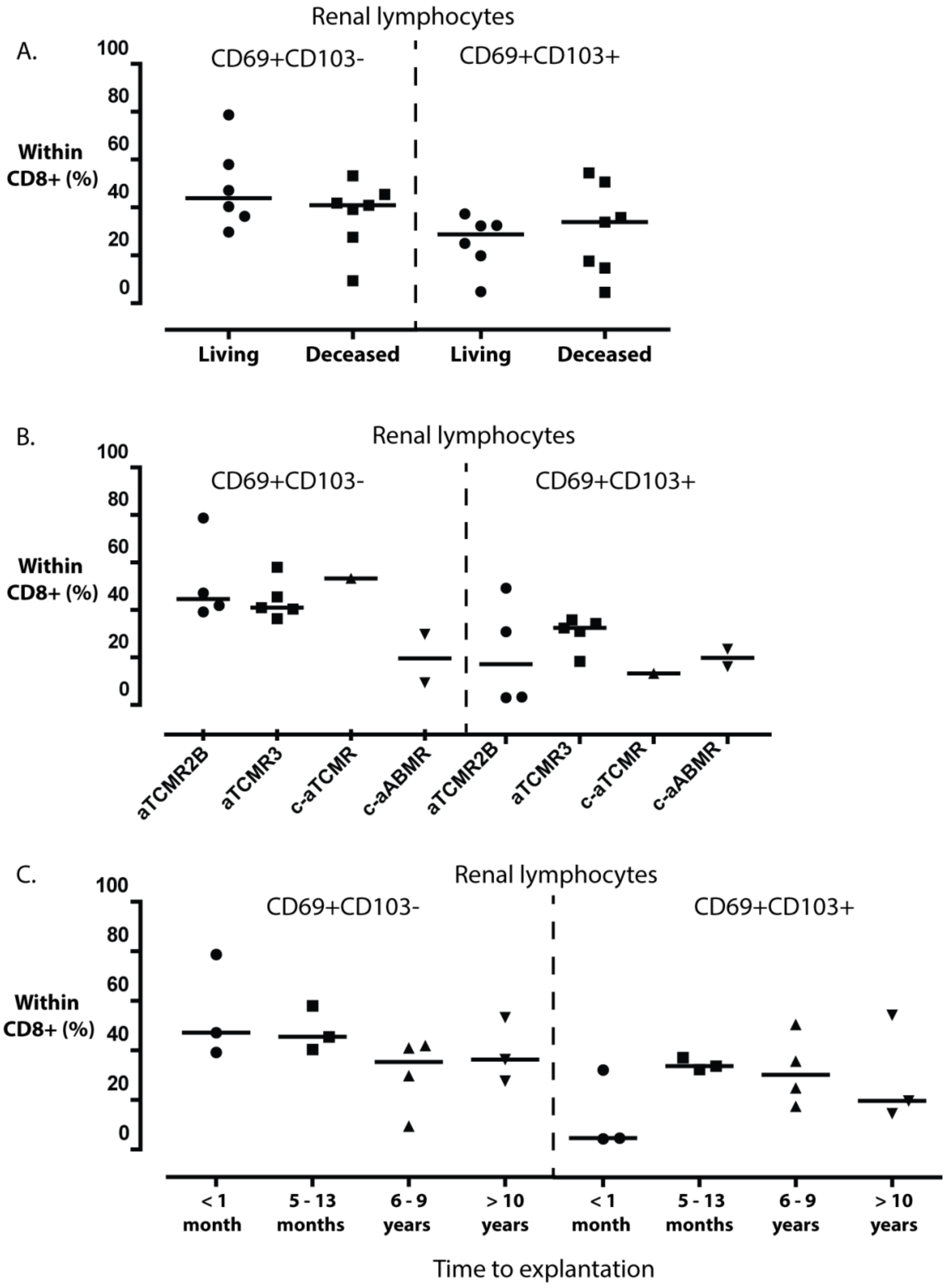

Supplemental figure 3.

No significant differences in T_{RM} cell frequencies when subdividing on donor type, Banff 2017 category, and time to explantation.

We subdivided the CD103- and CD103+ T cells within the CD8+ compartment based of donor type (A), Banff 2017 category (B), and time to explantation (C). No significant differences in T_{RM} cell frequencies were detected between the different subgroups.

Supplemental figure 4.

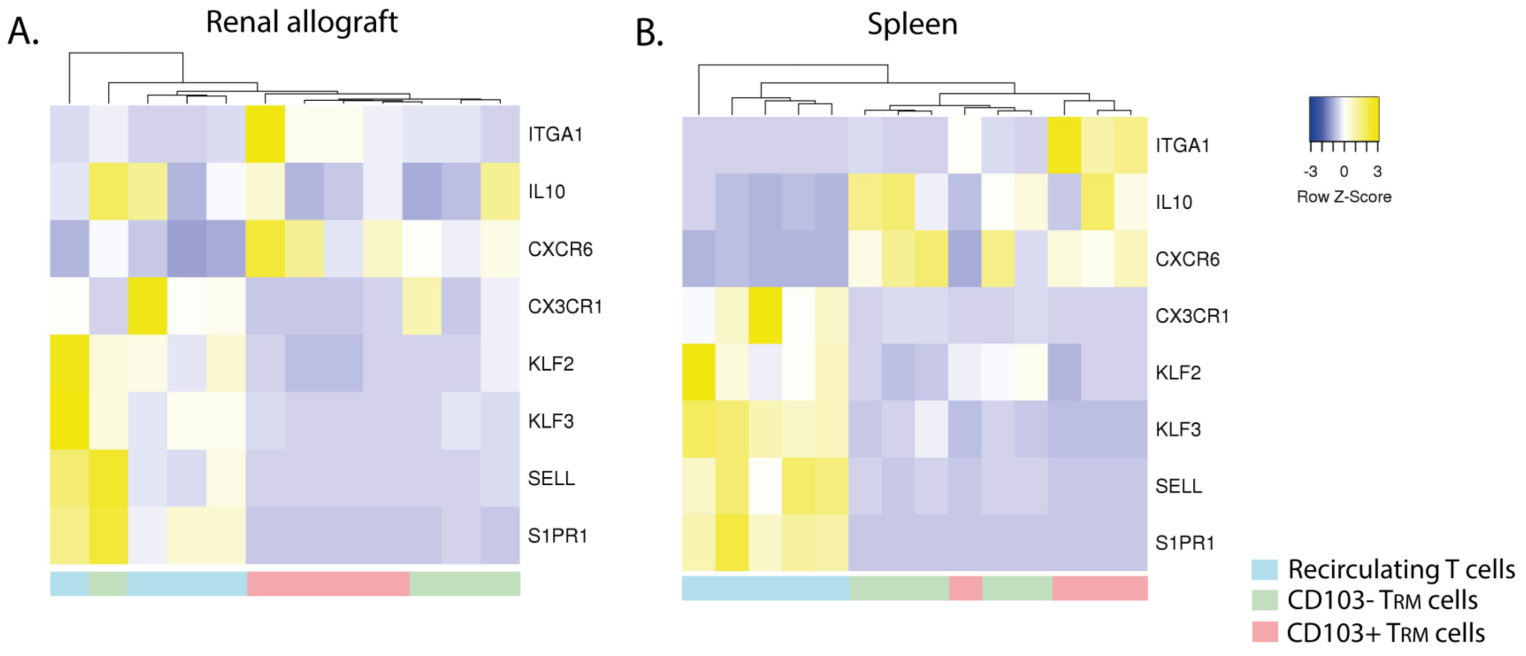

Supplemental figure 4.

Clustered expression of T_{RM} signature genes

Heatmaps depicting the normalized gene expression of eight core genes in the renal allograft (A) and spleen (B). The genes depicted are known to be upregulated (yellow) or downregulated (blue) in T_{RM} cells. Gene expression was measured in recirculating T cells (blue line), CD103- T_{RM} cells (green line), and CD103+ T_{RM} cells (red line) within the CD8+ compartment. Spleen *n* = 5, renal lymphocytes *n* = 4. Average linkage method was used to cluster the different samples and the Euclidean method was used to calculate the distance between the different samples

Supplemental figure 5.

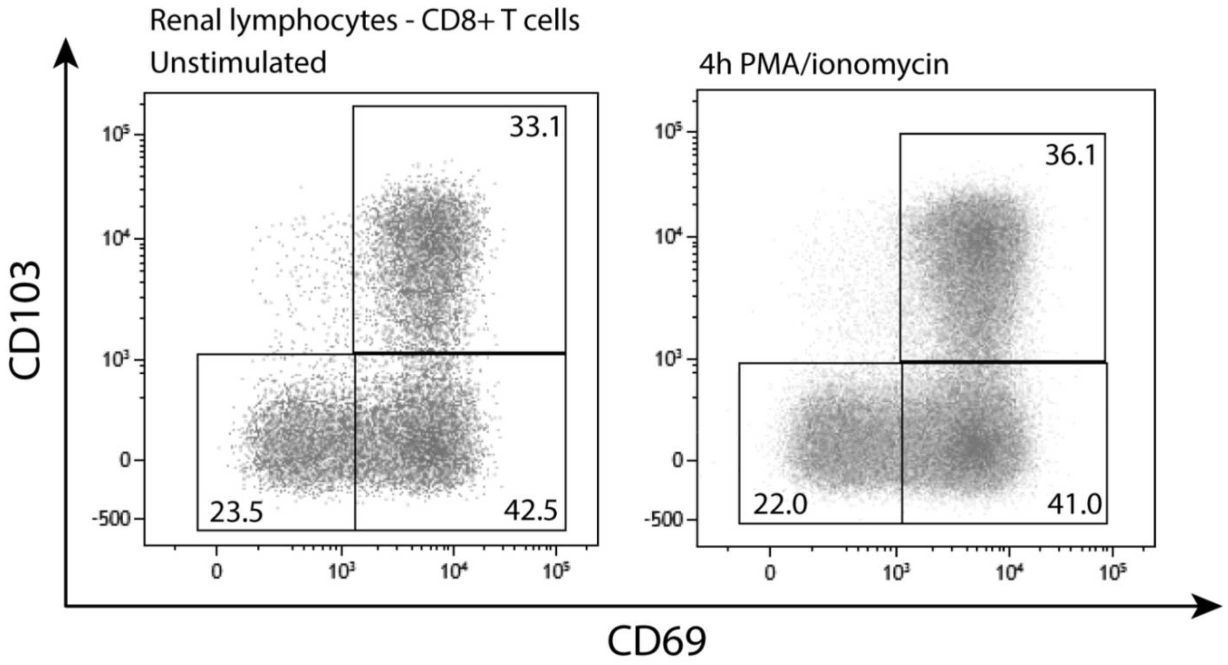

.

Supplemental figure 5.

No difference in CD69 and CD103 expression before and after PMA/ionomycin stimulation

Renal lymphocytes were stimulated for 4 hours with PMA/ionomycin in order to study cytokine producing capacities. Before and after stimulation the expression of CD69 and CD103 was measured with flow cytometry. Proportions of gated areas are depicted within the figure.

Supplemental figure 6.

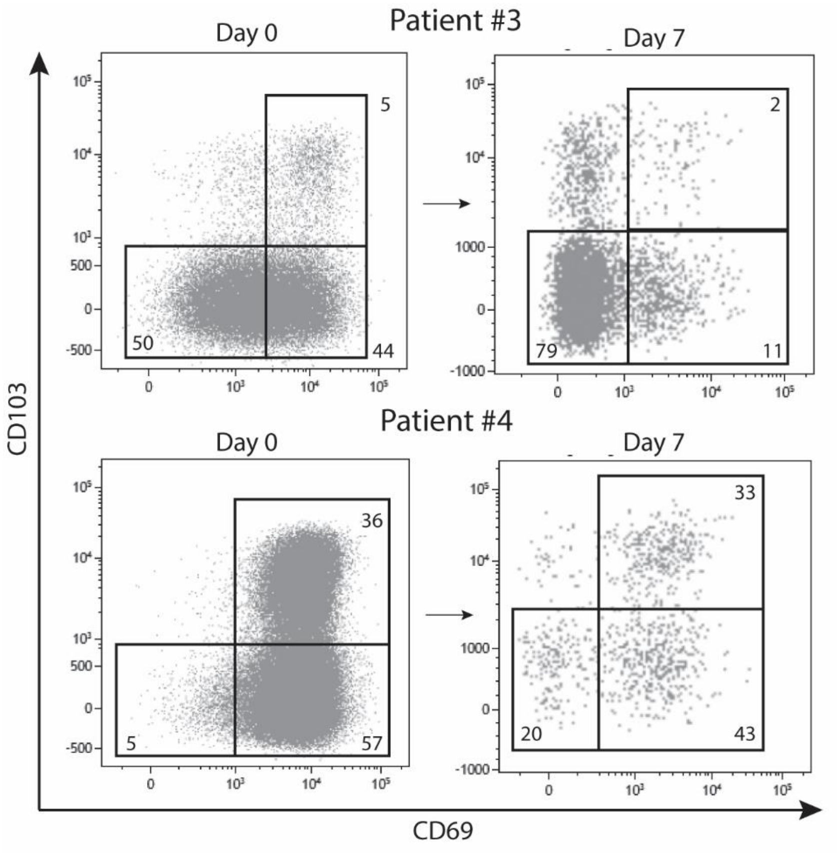

Supplemental figure 6.

Decreased expression of CD69 and CD103 on renal CD8+ T cells after allostimulation

Renal lymphocytes of patient number three and four (Table 1) were stimulated with the corresponding donor cells for seven days. Subsequently, cells were stained for CD8+ T cells. Within the CD8+ T cells, proportions of CD69 and CD103 were measured. Numbers in the figure represent the proportions of the gated cells.

Supplemental figure 7.

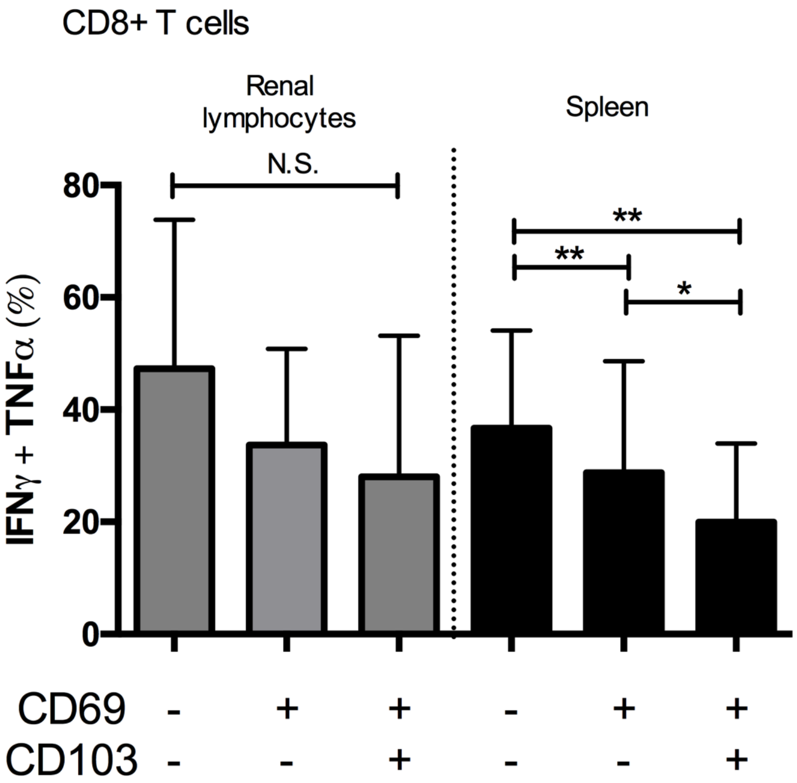

Supplemental figure 7.

Proportions of different T_{RM} subsets that produce both IFNγ and TNFα

Proportions of IFNγ and TNFα producing cells are depicted upon 4 hours PMA/ionomycin stimulation in the presence of monensin and brefeldin A. Cytokine proportions were measured in renal lymphocytes and splenocytes within the recirculating T cells, CD103- T_{RM} cells, and CD103+ T_{RM} cells of the CD8+ T cell compartment. Frequencies of positive cells were shown as mean with the SEM (renal lymphocytes n = 6, spleen n = 10). Significant differences were calculated and depicted (N.S. = not significant, * $p < 0.05$, ** $p < 0.01$ *** $p < 0.001$).
